# A deployment-oriented quality-control framework for integrating UAV LiDAR and field inventory in precision forestry

**DOI:** 10.3389/fpls.2026.1932005

**Published:** 2026-09-14

**Authors:** Xiangfei Lu, Ziyang Liu, Zheyuan Wu, Rui Cheng, Liyong Fu, Qingjie Wang, Xiaodi Zhao

**Affiliations:** 1 Research Institute of Forestry Policy and Information, Chinese Academy of Forestry, Beijing, China; 2 Institute of Forest Resource Information Technique, Chinese Academy of Forestry, Beijing, China; 3 Central South University of Forestry and Technology, College of Forestry, Changsha, China

**Keywords:** forest inventory, forest monitoring, quality control, UAV-LiDAR, uncertainty reporting

## Abstract

Reliable integration of UAV-LiDAR and field inventory is essential for operational forest monitoring, but heterogeneous data quality can destabilize plot-level predictions. Conventional quality-control procedures generally focus on filtering individual records or applying fixed pass–fail rules, providing limited support for differentiated decisions at the plot level. We developed a plot-level quality governance framework centred on QC_score. Unlike record-level screening or binary exclusion alone, QC_score combines spatial matching quality, field–LiDAR structural consistency, and data completeness into a continuous measure of plot-level risk, which is then used to prioritize review, determine plot admission, and support quality-adaptive uncertainty reporting. The framework also integrates staged quality control and low-cost repair assessment and was evaluated using 26,767 tree records from 133 plots in Guangdong Province, China. Prediction instability was driven mainly by plot-level structural inconsistency rather than isolated tree-level errors. Excluding structurally inconsistent plots outperformed both full retention and soft quality weighting. For dominant plot height, hard exclusion after QC.3 reduced RMSE from 1.927 to 1.421 m. In the separate cost-based admission analysis, a QC_score threshold of 0.579 retained 95 plots and yielded an RMSE of 1.146 m. In a separate quick-repair experiment, QC123-hard reduced RMSE from 2.597 to 1.459 m relative to the QC12 baseline. Mondrian conformal calibration achieved more balanced coverage across quality grades while assigning narrower intervals to higher-quality plots. The framework therefore provides auditable rules for plot admission, anomaly handling, and uncertainty-aware deployment of UAV-LiDAR forest inventory products.

## 1 Introduction

The global environment is under increasing pressure, and forests play a central role in climate-change mitigation and ecological sustainability (Forzieri et al., 2022). Forests are a key component of the terrestrial carbon sink and constitute an important pillar of nature-based climate solutions (Mo et al., 2023). However, ongoing human activities and multiple disturbance regimes continue to drive forest loss and degradation, thereby threatening the capacity of forests to sustain carbon storage, biodiversity, and other ecosystem services (Lechner et al., 2020; Bullock et al., 2022). Maintaining and enhancing forest ecological functions therefore depend on effective silviculture, management, protection, and utilization, all of which require accurate, timely, and reliable forest resource monitoring (Curtis et al., 2018; Buma et al., 2024).

For management-oriented forest inventory, the usefulness of monitoring outputs is determined not only by predictive accuracy under experimental settings, but also by whether the information remains robust, validated, and operationally usable in real planning contexts (Ulvdal et al., 2023). Forest inventory data serve as the core information base for hierarchical planning, and uncertainty in such data can propagate into management outcomes and objective fulfilment (Janová et al., 2024; Ulvdal et al., 2025). Forest monitoring therefore requires not merely high-accuracy predictions for selected plots, but reliable information products that support planning, updating, and decision-making under real-world deployment conditions (Holopainen et al., 2010; Eyvindson et al., 2017). In this sense, deployment reliability and practical usability are as important as model accuracy itself.

In recent years, UAV-LiDAR has become an important tool for forest inventory because of its ability to efficiently capture three-dimensional forest structure (Spiers et al., 2025). The extraction of individual-tree structural attributes from point clouds and the inversion of plot-level parameters have become key technical pathways for precision forest monitoring (Puliti et al., 2020). Compared with conventional field surveys, UAV-LiDAR offers higher acquisition efficiency, stronger spatial continuity, and greater capacity to characterize forest structure (Corte et al., 2020; Jaskierniak et al., 2021; Ramalho de Oliveira et al., 2021). It can provide rich information on canopy height, crown size, canopy volume, and vertical stand structure, thereby supporting the transition of forest surveys from manual sampling toward more digital, detailed, and frequent observation (Fekry et al., 2022).

In operational applications, however, integrating UAV-LiDAR with field inventory is not simply a matter of linking two datasets (Persson et al., 2022; Janssen et al., 2023). Its performance depends on accurate co-registration between field plots and LiDAR data, high-quality field reference data, and consistency between LiDAR-derived structural metrics and field measurement protocols (Wang et al., 2019). Plot-location errors and other co-registration mismatches can alter LiDAR canopy metrics, regression coefficients, and prediction accuracy, while field-reference errors—including measurement, model, and position errors—can bias model calibration and inflate apparent prediction error (Hauglin et al., 2021). In addition, project-specific differences in sensor settings, acquisition geometry, scan angle, and processing workflow can introduce systematic between-project effects that reduce the reliability of wall-to-wall predictions (Frazer et al., 2011; Dayal et al., 2022). These problems are therefore not merely tree-level noise; they can propagate to plot-level estimates, distort stand relationships, undermine prediction reliability, and ultimately reduce the usefulness of monitoring products for inventory updating and forest management decision-making (Ruotsalainen et al., 2021).

However, most existing studies have been conducted under relatively controlled data conditions, with primary attention devoted to algorithm development, model benchmarking, and predictive performance comparison, whereas substantially less emphasis has been placed on the quality, representativeness, and operational fitness of input data in real deployment settings (Hamedianfar et al., 2022; Fassnacht et al., 2024). Compared with experimental datasets, operational forest inventory data are typically more heterogeneous and are exposed to more complex error sources, including plot-location and co-registration errors, outliers and protocol-related inconsistencies, missing or erroneous reference values, temporal or spatial mismatch, and differences in measurement or processing workflows across projects (Persson et al., 2022; Knott et al., 2023; Lisańczuk et al., 2024). These issues may not appear as obvious anomalies at the individual-tree level, but they can propagate after aggregation to the plot or stand scale, thereby affecting model training, uncertainty behaviour, and deployment reliability (Kotivuori et al., 2021; Fassnacht et al., 2024). In other words, the main bottleneck in operational UAV-LiDAR and field-inventory integration is often not simply whether a model is accurate, but whether the input data are trustworthy, the risks are diagnosable, and the resulting products are interpretable and usable for management (Fassnacht et al., 2024).

Most previous studies have concentrated on improving individual-tree segmentation, attribute extraction, and predictive accuracy, whereas substantially less attention has been devoted to how data quality, uncertainty characterization, and quality assurance should be handled for operational forest inventory use (White et al., 2016; Persson and Ståhl, 2020; Xiu et al., 2025). For management applications, however, the more consequential questions are often which data-quality problems actually compromise plot-level use, how problematic plots should be screened or flagged, and how uncertainty should be communicated in a form that is usable for inventory delivery and decision support. In operational forestry, uncertainty in remotely sensed inventory attributes can propagate into management outcomes and economic losses, while insufficient QA/QC can compromise the credibility of the information delivered to managers and decision makers (Brandeis et al., 2017; Candelas Bielza et al., 2026). For deployment-oriented forest monitoring, these questions are at least as important as model performance itself. This is particularly true at the plot scale, where a small number of structurally inconsistent plots can exert disproportionate influence on model training and inference. Quality control in forest monitoring should therefore not be treated simply as a routine preprocessing step, but as part of an explicit quality-governance framework that supports plot admission, risk stratification, anomaly review, and uncertainty reporting (Brandeis et al., 2017; Persson and Ståhl, 2020; Candelas Bielza et al., 2026).

Against this background, this study develops a plot-level quality-governance framework for integrating UAV-LiDAR and field inventory in forest monitoring. Rather than treating quality control as routine preprocessing, we conceptualize it as an operational workflow for identifying deployment-relevant anomalies, stratifying plot-level risk, supporting plot screening and targeted correction where feasible, and reporting uncertainty in a manner aligned with data quality. This study makes three main contributions (Pause et al., 2016; Junttila and Kauranne, 2018; Karppinen et al., 2024; White et al., 2025). First, it shifts the focus of UAV-LiDAR and field-inventory integration from model-centred accuracy improvement toward plot-level quality governance for operational forest inventory. Second, it distinguishes tree-level contamination from plot-level structural inconsistency and shows that the latter is the more consequential source of deployment failure and therefore requires explicit handling rules. Third, it translates quality diagnostics into actionable procedures for plot screening, anomaly review, and uncertainty-aware use, thereby improving the reliability and management usability of UAV-LiDAR-assisted forest inventory.

## 2 Data and methods

### 2.1 Study design and data sources

This study was designed to support the operational integration of UAV-LiDAR-derived individual-tree metrics with field inventory data under real-world conditions. The analysis focused on data-quality governance and model generalization. The dataset comprised 26,767 individual-tree records linked between field measurements and LiDAR observations across 133 plots. All analyses were conducted at the plot level, with plots treated as the statistically independent unit, to avoid overestimating generalization performance due to pseudo-replication among tree-level samples. The study area was located in Guangdong Province, China, where Chinese fir (*Cunninghamia lanceolata*) was the dominant tree species (**Figure 1**).

**Figure 1:**
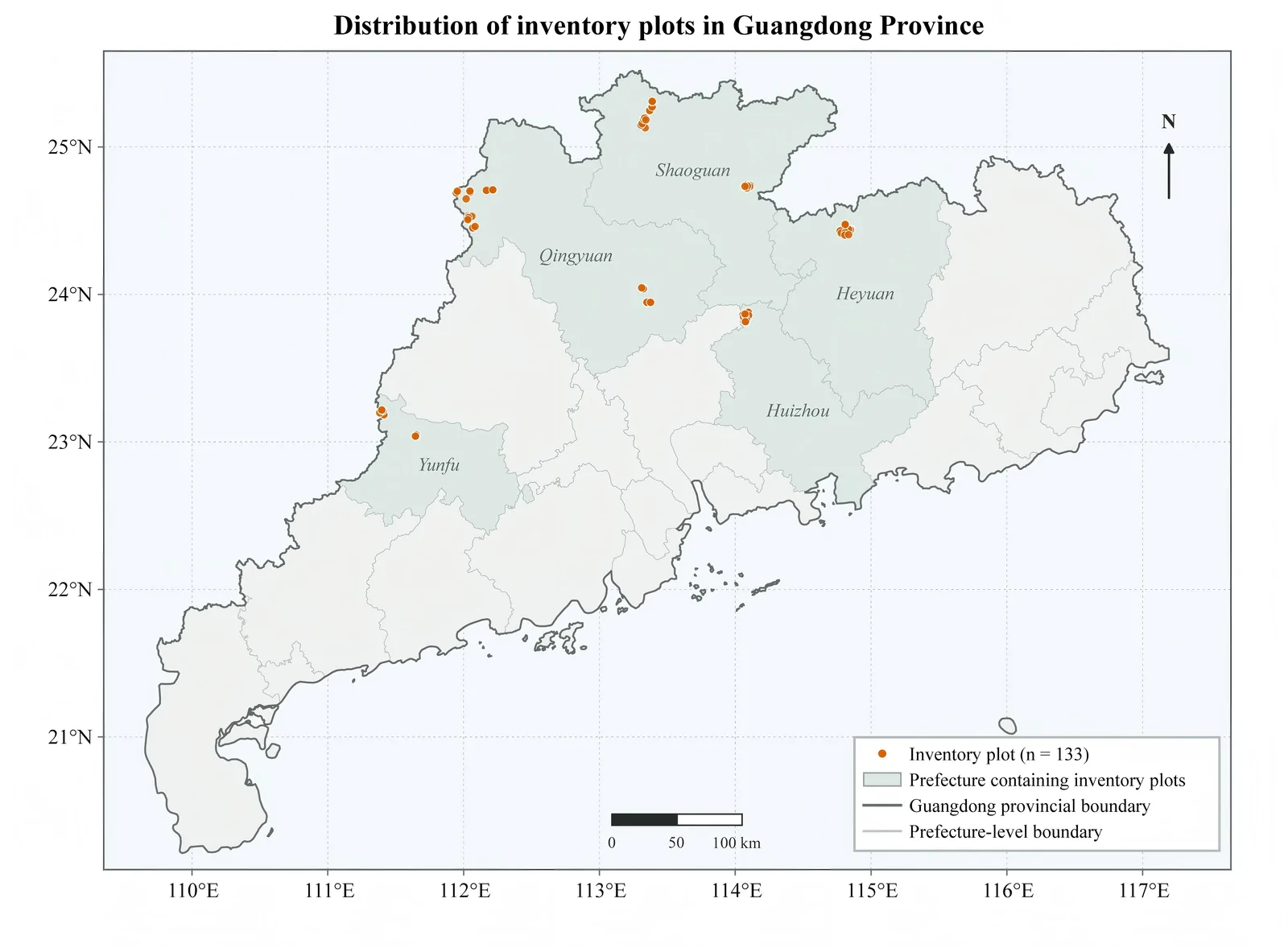
Study area.

UAV-LiDAR data were acquired in March 2024 using a BB4 unmanned aerial vehicle equipped with an AS-1300HL laser scanning system. The scanner was a Riegl VUX-1LR, with a wavelength of 1550 nm, a pulse length of 3.5 ns, and a beam divergence of 0.5 mrad. The pulse repetition rate was 50 kHz, the maximum scan angle was 30°, and the scan frequency was 49 Hz. Flights were conducted along a grid-pattern trajectory, with a side-overlap rate of 50% and an average flight speed of 10 m s^−1^. The mean point-cloud density across plots was 110 points m^−2^.

The field inventory included core individual-tree attributes, including tree location (X and Y), diameter at breast height (DBH), tree height (H), and crown base height (CBH), together with selected plot-level background information such as stand origin, age class, management unit, and topographic conditions. Because operational data collected across multiple batches, personnel, and devices are prone to inconsistent coordinate conventions, contamination from missing-value coding, and batch-specific variation, we implemented an explicit three-stage quality-control procedure before modeling and derived a plot-level quality score (QC_score) to support risk stratification and review triggering.

### 2.2 Point-cloud processing, individual-tree metric extraction, and tree-location output

UAV-LiDAR point-cloud preprocessing included noise and outlier filtering, ground-point classification, digital elevation model (DEM) generation, and height normalization (Yu et al., 2024). Normalized height was calculated using Equation 1:

(1) $$z_{norm}=z-DEM$$

Based on the normalized point cloud, a canopy height model (CHM) was generated at a spatial resolution of 0.2 m. To reduce interference from residual ground noise and low vegetation during individual-tree segmentation, a minimum height threshold of z_norm ≥ 0.2 m was applied in the main workflow (You et al., 2023; Yusup et al., 2025).

Individual-tree segmentation was conducted using a self-developed deep-learning algorithm for three-dimensional point-cloud segmentation. The model achieved a recall of approximately 0.99, an F1 score of 72%, an intersection over union (IoU) of 75%, and an overall accuracy (OA) of 78%. Because the segmentation model was not trained or fine-tuned using the 133 plots analyzed in this study, the tree-level segmentation procedure did not incorporate information from the plot-level prediction experiments. After segmentation, LiDAR-derived individual-tree metrics and tree locations were extracted for each tree. The main metrics included tree height ( TreeHeight, m), crown diameter ( CrownDiameter, m), crown area ( CrownArea, m²), and crown volume ( CrownVolume, m³). The output tree coordinates $\left(X_{L},Y_{L}\right)$ were used for spatial matching with field-recorded tree locations (Douss and Farah, 2022).

### 2.3 Tree matching under one-to-one constraints

In operational datasets, mixed coordinate fields are common and can lead to systematic mismatches during tree-level spatial matching. Typical problems include swapped X/Y coordinates in field records, inconsistent field definitions, and projection-related errors. Such issues can substantially reduce the validity of plot-level structural features and introduce biases that are difficult to interpret. To address this problem, we explicitly incorporated coordinate-consistency correction and dual-interpretation distance into the tree-matching procedure (Dorigo et al., 2010; Lamprecht et al., 2017; Fadili et al., 2019). Using the LiDAR-derived tree coordinates as the reference, we calculated the Euclidean distance for each field-recorded tree under two alternative coordinate interpretations, as shown in Equations 2 and 3:

(2) $$d_{XY}=\sqrt{\;{\left(X-X_{L}\right)}^{2}+{\left(Y-Y_{L}\right)}^{2}}$$

(3) $$d_{YX}=\sqrt{\;{\left(Y-X_{L}\right)}^{2}+{\left(X-Y_{L}\right)}^{2}}$$

If $d_{XY}<d_{YX}$, the original interpretation was retained, such that $\left(X^{*},Y^{*})=(X,Y\right)$; otherwise, the swapped interpretation was used, such that $\left(X^{*},Y^{*})=(Y,X\right)$. The final matching distance was then defined using Equation 4:

(4) $$d=\min(d_{XY},d_{YX})$$

For each field-recorded tree, candidate matches were searched within the LiDAR tree set using a radius of r=10 m. Candidate pairs with the smallest matching distance were retained for subsequent conflict resolution, and the final matching distance *d* was recorded as a core quality indicator for the first-stage quality check (Monnet and Mermin, 2014; Pascual, 2019).

To avoid pseudo-matching caused by one LiDAR tree being linked to multiple field trees, or vice versa, a one-to-one constraint was imposed on the candidate pairs. Specifically, all candidate pairs were sorted in ascending order of *d*, and pairs were greedily selected only when neither the field tree nor the LiDAR tree had been assigned previously. Candidate pairs were skipped if either side had already been occupied. This procedure is equivalent to an approximate implementation of one-to-one assignment, and the same strategy was applied consistently throughout the analysis. For each successful match, the output fields included the matching distance *d*, the matched-pair identifier, and diagnostic information such as conflict counts.

In the main analysis, records with d>5 m were treated as suspected mismatches and excluded. Additional thresholds of 3 and 10 m were used for sensitivity analysis to test the robustness of the results to the strength of matching-distance gating. Matching quality was reported transparently in the Results section using summary statistics of the distance distribution (e.g., $d_{50}$ and $d_{95}$), changes in record retention and plot coverage under alternative thresholds, and the resulting effects on key modeling outcomes.

### 2.4 Response variables, predictors, and plot-level aggregation

#### 2.4.1 Response variables

Plot-level height metrics were used as the primary response variables in this study. Although tree-level records were numerous, the main source of statistical independence lay at the plot level. Direct regression at the tree level would therefore risk pseudo-replication and could lead to overestimation of model generalization. Accordingly, the analysis focused on the following two plot-level response variables.

The first response variable was mean plot height, defined using Equation 5:

(5) $${\bar{H}}_{p}=\frac{1}{N_{p}}\sum_{i=1}^{N_{p}}H_{ip}$$

where $H_{ip}$ is the field-measured height of the *i*-th tree in plot *p*, and $N_{p}$ is the number of trees with valid height measurements in that plot.

The second response variable was dominant plot height, defined as the mean height of the tallest 20% of trees within each plot using Equation 6:

(6) $$H_{dom20,p}=\frac{1}{K_{p}}\sum H_{pj}$$

where the summation is taken over the $K_{p}\;tallest\;valid\;trees\;in\;plot\;p$. $H_{pj}$ denotes the field-measured height of tree *j* in plot *p*, and *K_p_* is calculated as:


$$K_{p}=[0.2N_{p}]$$

where $N_{p}$ denotes the number of valid tree-height observations in plot p. Here, floor(·) denotes rounding down to the nearest integer. This convention ensures that the number of trees used to calculate dominant plot height does not exceed 20% of the valid trees in a plot and provides a deterministic rule when 20% of the tree count is not an integer.

#### 2.4.2 Predictors

Predictors were grouped into three categories: LiDAR-derived structural features (F1), topographic variables added to F1 (F2), and management/background variables further added to F2 (F3). All tree-level LiDAR metrics were aggregated to plot-level statistics, and heavy-tailed variables were transformed using robust functions to improve model stability. The response variables, predictors, and feature-set definitions used for plot-level modeling are summarized in **Table 1**.

**Table 1:** Variables and feature sets used for plot-level modeling.

Variable	Level	Unit	Source
H_mean_field	Plot	m	Field inventory
Dominant plot height (H_dom20_field)	Plot	m	Field inventory
TreeHeight (m)	Tree	m	UAV-LiDAR
CrownDiameter	Tree	m	UAV-LiDAR
CrownArea	Tree	m²	UAV-LiDAR
CrownVolume	Tree	m³	UAV-LiDAR
Tree density	Plot	Trees·ha^-1^	UAV-LiDAR
Canopy cover proxy (Cover)	Plot	%	UAV-LiDAR
Crown volume density (VD)	Plot	m³/m²	UAV-LiDAR
Structural heterogeneity	Plot	–	UAV-LiDAR
Elevation	Plot	m	DEM/Topo
Slope	Plot	degree	DEM/Topo
Aspect (sin, cos)	Plot	–	DEM/Topo
Origin	Plot/Compartment	–	Inventory/Management
Age class	Plot/Compartment	–	Inventory/Management
Management unit	Compartment	–	Inventory/Management
Canopy closure	Plot/Compartment	%	Inventory/Management
Mean stand age	Plot/Compartment	years	Inventory/Management

F1 includes LiDAR-derived structural predictors only; F2 adds topographic variables; F3 further adds management-related variables. Structural predictors include canopy cover proxy and crown volume density at the plot level.

##### 2.4.2.1 LiDAR-derived structural features (F1)

Tree-level LiDAR metrics were aggregated to the plot scale to characterize stand structure. Height-related features included the mean, standard deviation, maximum, and 95th percentile of TreeHeight. Crown-related features included the sum and mean of CrownVolume, as well as the mean of CrownDiameter. Density- and cover-related proxies included LiDAR-derived tree density $\left(N_{\text{ha}}\right)$, canopy cover proxy (Cover), and crown volume density (VD).

To further characterize stand structural complexity and heterogeneity, three derived metrics were constructed. The coefficient of variation in tree height was defined using Equation 7:

(7) $$C_{VH}=\frac{sd(TreeHeight)}{mean(TreeHeight)}$$

The interquantile height range defined using Equation 8:

(8) $$D_{H}=p95(TreeHeight)-p50(TreeHeight)$$

and upper-canopy dominance was defined using Equation 9:

(9) $$R_{H}=\frac{p95(TreeHeight)}{mean(TreeHeight)}$$

To facilitate interpretation for management applications, a canopy cover proxy was calculated using Equation 10:

(10) $$Cover=\frac{\sum CrownArea}{A_{p}}$$

Crown volume density was calculated using Equation 11:

(11) $$VD=\frac{\sum CrownVolume}{A_{p}}$$

and LiDAR-derived tree density was calculated using Equation 12:

(12) $$N_{ha}=\frac{N_{p}}{A_{p}}\times 10^{4}$$

where $A_{p}\;$ is the plot area. For heavy-tailed variables such as CrownArea, CrownVolume, and VD, the main analysis applied a log(1+x)  transformation to reduce the influence of extreme values. When canopy overlap resulted in Cover>1, this variable was interpreted as canopy projection intensity rather than literal areal coverage; in such cases, logarithmic transformation was used in the main analysis, and robustness was further examined in sensitivity analyses.

##### 2.4.2.2 Topographic variables (F2 added to F1)

Topographic variables included slope, aspect, and elevation. Aspect was encoded using sine and cosine transformation (sinθ,cosθ), where θ is the aspect angle. When aspect was recorded using Chinese directional labels or mixed coding formats, it was first standardized and mapped to a consistent angular representation before encoding.

##### 2.4.2.3 Management and background variables (F3 added to F2)

Management and background variables included stand origin, age class, management compartment, canopy closure, and mean stand age. Categorical variables were encoded using one-hot encoding, and numerical variables were standardized within each training fold.

### 2.5 Three-stage quality control and plot-level quality scoring

To systematically reduce the effects of mismatches, coding contamination, physically implausible values, and plot-level inconsistencies in height measurement systems that are common in operational data, we implemented a three-stage quality-control procedure (QC.1–QC.3). In addition, a continuous plot-level quality score, denoted as QC_score and scaled from 0 to 1 (with higher values indicating better data quality), was constructed to support risk stratification and review triggering in deployment. The overall workflow produced two datasets for modeling and robustness assessment: QC123-hard as the main analysis set and QC123-full as the sensitivity-analysis set. The QC12 dataset, in which no plot-level exclusion was applied, was retained as a comparison baseline.

#### 2.5.1 Matching-quality gating (QC.1)

In the first stage, the tree-level spatial matching distance *d* was used as the indicator of matching quality. In the main analysis, tree records with d>5m were excluded to reduce contamination from mismatches in the aggregation of structural features and subsequent model training. Additional thresholds of 3 and 10 m were used for sensitivity analysis, and the corresponding record-retention rates, plot coverage, and error metrics were reported transparently to evaluate the robustness of the conclusions to gating strength. The threshold choice was guided by the need to balance the exclusion of serious mismatches against the retention of sufficient plots and sample size under operational conditions.

#### 2.5.2 Missingness and coding consistency (QC.2)

This stage adopted a conservative strategy of setting values to missing or flagging them as anomalous, without smoothing or replacing valid observations. Only values that were clearly physically implausible or known to represent missing-code contamination were standardized as missing, so as to reduce label noise and distortions in derived variables. The rules were as follows.

First, missingness and physical constraints were checked. Non-positive values of DBH and H (e.g., DBH ≤0, H ≤0) were treated as missing. Crown base height (CBH) was subject to physical-constraint checks, and values with CBH<0 or CBH>H were treated as missing. Non-positive values of LiDAR-derived tree height (TreeHeight) were also treated as missing to avoid contamination of aggregated height-structure features.

Second, missing-code consistency and recalculation were addressed. When plot-level height fields contained “0” values used as missing codes, these were uniformly treated as missing. Mean plot height and dominant plot height were then recalculated from valid tree-level observations according to the predefined response-variable definitions (Section 2.4.1), in order to prevent zero-coded labels from entering model training and evaluation.

Third, robust transformation was applied to heavy-tailed variables for modeling stability without altering the original observations. For variables such as CrownVolume, CrownArea, and VD, a log(1+x) transformation was applied before modeling. This transformation was fitted within each training fold of cross-validation (Section 2.6.1) to avoid information leakage.

#### 2.5.3 Plot-level consistency diagnostics and two-level exclusion (QC.3)

After obtaining relatively reliable tree-level samples through QC.1 and QC.2, QC.3 evaluated the consistency between field-measured tree height *H* and LiDAR-derived TreeHeight at the plot level in order to identify plots with inconsistent height systems or structurally abnormal noise that were most damaging to deployment. For each plot *p*, based on valid matched pairs with both *H* and TreeHeight available, the mean height difference (systematic bias) and root mean square error were calculated using Equations 13 and 14, respectively:

(13) $$\Delta {\bar{H}}_{P}=\frac{1}{N_{p}}\sum (TreeHeight_{ip}-H_{ip})$$

(14) $$RMSE_{p}=\sqrt{\frac{1}{N_{p}}\sum {\left(TreeHeight_{ip}-H_{ip}\right)}^{2}}$$

and the correlation coefficient was calculated using Equation 15:

(15) $$corr_{p}=corr(H,TreeHeight)$$

A minimum paired-sample constraint of $N_{p}\geq 5$ was imposed to avoid unstable statistics.

To reduce the disproportionate influence of a few extreme plots on threshold estimation, robust outlier thresholds were defined using the median and median absolute deviation (MAD). For a given plot-level diagnostic variable x(e.g., $RMSE_{p}$, and in the full scenario also $|\Delta {\bar{H}}_{p}|$), the anomaly threshold was defined using Equation 16:

(16) T=median(x)+3×MAD(x)

Where MAD(x)=median(|x−median(x)|)This thresholding rule is robust and distribution-insensitive, and is less easily distorted by heavy-tailed distributions or occasional measurement failures than conventional mean–standard-deviation rules, thereby improving the stability of plot screening under operational noise conditions.

Based on these diagnostics, two QC.3 datasets were constructed to balance deployability and sensitivity analysis. The main analysis used the QC123-hard dataset, in which a plot was flagged as anomalous and excluded if any of the following conditions was met: (i) $RMSE_{p}>T_{RMSE}$; (ii) $corr_{p}\leq 0$; (iii) The number of valid matched pairs was fewer than five. This dataset preferentially removed plots with severely distorted height consistency or insufficient statistical information, thereby improving the stability and interpretability of model training and deployment.

The sensitivity analysis used the stricter QC123-full dataset. In addition to the exclusion rules in QC123-hard, this dataset also excluded plots that showed excessive systematic bias, defined as $|\Delta {\bar{H}}_{p}|>T_{|\Delta H|}$. This rule was intended to identify plots that retained acceptable correlation but showed substantial systematic offset, and to test whether the study conclusions changed materially under a more conservative bias-exclusion strategy.

#### 2.5.4 Plot-level quality score and quality stratification

To extend quality control from rule-based exclusion to a deployable quantitative quality system, a plot-level quality score, $QC\_score_{p}$, was constructed. For each plot, quality-related features were calculated to represent matching quality (e.g., $d_{p,95}$ or $d_{p,50}$), consistency ( $RMSE_{p}$, $|\Delta {\bar{H}}_{p}|$, and $corr_{p}$), and completeness (e.g., the proportion of valid height observations and the anomaly rate). Each metric was robustly standardized to the range [0,1], with 1 indicating better quality and 0 indicating poorer quality, and then aggregated using equal or fixed weights according to Equation 17:

(17) $$QC_{score_{p}}=\sum_{j=1}^{J}w_{j}z_{pj},\sum w_{j}=1$$

where $z_{pj}\in [0,1]$ is the standardized score of the j-th quality indicator for plot p, and $w_{j}$ is its corresponding weight. Equal weighting was adopted in the main analysis to minimize uncertainty introduced by subjective weighting choices. In extended analyses, the weights could be adjusted to emphasize specific operational risks, such as mismatch risk or inconsistency in height systems.

The quality score was further used for plot-level stratification and threshold learning. Specifically, QC_score was used to define three quality grades—High, Medium, and Low—based on terciles, denoted as QC_grade. Using the high-error event defined in Section 3 as an external reference, thresholds were then learned using ROC/Youden analysis or cost-sensitive criteria, thereby forming deployment-oriented review-triggering rules; for example, plots with QC_score<Tcould be assigned to a review or reprocessing queue.

### 2.6 Deployment-oriented evaluation framework

To ensure that model outputs were suitable for deployment and free from data leakage, all modeling and evaluation were conducted at the plot level, with plots treated as the independent analytical unit. Feature processing, model training, threshold learning, and interval calibration were all performed within a cross-validation framework. Generalization error and uncertainty were then reported under multiple validation settings designed to better approximate operational deployment conditions.

#### 2.6.1 Modeling scenarios and leakage control

To support deployment-oriented interpretation while avoiding data leakage, two modeling scenarios were defined and trained separately.

##### 2.6.1.1 Pure LiDAR deployable model.

The main conclusions were based only on predictors that could be obtained directly from UAV-LiDAR data, including structural features derived from tree height and crown metrics, density and cover proxies, and structural heterogeneity indicators, together with wall-to-wall topographic or background variables such as slope, sine- and cosine-transformed aspect, elevation, canopy closure, mean stand age, and stand origin. Variables derived from field-measured DBH or tree height, or variables that depended strongly on field inventory intensity (e.g., basal area, quadratic mean diameter, or height-to-diameter ratio), were excluded to ensure reproducibility and transferability under operational conditions where only point clouds and basic background information are available.

##### 2.6.1.2 Semi-assisted inventory model (extended analysis)

As an extended analysis, the main scenario was supplemented with auxiliary information that could plausibly be obtained at relatively low cost in management practice, such as historical summary indicators or simple statistics derived from rapid measurements in a limited number of plots. This scenario was used only to evaluate the marginal benefit of small amounts of additional information and to help interpret error sources; it was not used in place of the main conclusions.

To obtain unbiased estimates of generalization performance, a strict leakage-control principle was applied consistently across all models and experimental settings. Within each outer cross-validation split, all data-driven preprocessing steps and decision rules were fitted using the training folds only, and were then applied to the corresponding test fold without further updating. Test data were not involved in any stage of model fitting, preprocessing calibration, or threshold learning. Specifically, missing-value handling (e.g., imputation based on the training-fold median or mode, with or without missingness indicators), feature scaling (e.g., standardization or normalization), monotonic transformation (e.g., log(1+x)), and categorical encoding (e.g., one-hot encoding or target encoding) were all parameterized within the training folds and then mapped to the test fold using the fitted parameters.

In addition, when experiments involved QC_score-based gating or risk stratification, the corresponding thresholds—including ROC/Youden-based thresholds and cost-sensitive operating points—were learned entirely within the training folds and then fixed for evaluation on the test fold. This design effectively avoided optimistic bias caused by allowing the test data to influence preprocessing or threshold selection, thereby providing a more conservative and more credible estimate of model performance under real deployment conditions.

#### 2.6.2 Role of the main model and comparison across model families

In the model-comparison experiments, a main model was specified. Its role was not to maximize predictive flexibility or achieve the most complex functional form, but to provide a controlled and auditable baseline framework for all subsequent analyses related to quality governance. Specifically, the main model served as the common reference for quantifying the benefits of quality control, defining gating thresholds, evaluating learning curves, testing spatial extrapolation, constructing prediction intervals, and supporting review-triggering mechanisms. Accordingly, the main model was required to satisfy four criteria. First, stability was prioritized: under a limited plot sample size (n = 122 in the main analysis set QC123-hard) and variable data quality, the model should show relatively low variance, controlled sensitivity to noise and outliers, and reproducible outputs. Second, it should be transparent and interpretable, so that the direction and contribution of predictors can be clearly examined for operational auditing, error tracing, and long-term maintenance. Third, it should be easy to maintain and recalibrate, with a limited number of hyperparameters and low training cost, making it suitable for routine updating and rapid recalibration. Fourth, it should be compatible with the QC workflow, such that the effects of quality-control rules, gating, and repair could be directly reflected in performance metrics and interpreted under a common reference framework.

Based on these considerations, ridge regression was selected as the main model for the primary analysis. Two additional model families were included for comparison: the generalized additive model (GAM), which was used to capture smooth nonlinear effects, and the gradient boosting decision tree (GBDT), which was used to capture more complex nonlinear relationships and higher-order interactions. These comparison models were introduced to evaluate whether added model complexity could provide stable and transferable gains. It should be emphasized, however, that GAM and GBDT were used only for supplementary validation and comparative assessment; they were not intended to replace the main model in the deployment-oriented analysis.

#### 2.6.3 Cross-validation design and evaluation metrics

The primary evaluation was based on repeated nested K-fold cross-validation at the plot level. In the outer loop, 30 folds were used and repeated R times. Within each outer-loop training set, hyperparameters were tuned using five-fold cross-validation in the inner loop. Model performance was evaluated using RMSE, MAE, $R^{2}$, bias, and relative RMSE (rRMSE). Bias and rRMSE were calculated using Equations 18 and 19, respectively:

(18) $$bias=\frac{1}{M}\sum_{p=1}^{M}({\hat{y}}_{p}-y_{p})$$

(19) $$rRMSE=\frac{RMSE}{\bar{y}}$$

In addition to random K-fold cross-validation, two additional robustness-oriented validation schemes were implemented to approximate a more conservative lower bound of deployment performance, that is, settings in which the test data were more challenging and exhibited stronger distribution shift than under random splitting. First, a leave-one-group-out (LOGO) validation was conducted based on age class. In each run, one age-class group was held out as the test set, and all remaining groups were used for training. This design directly evaluated model transferability and robustness across different stand developmental stages.

Second, to approximate a cross-operational-area extrapolation scenario, spatial block cross-validation was implemented. Plots were grouped into a number of spatial blocks according to their geographic locations, for example using coordinate-based *k*-means clustering. In each run, one spatial block was held out as the test set and the remaining blocks were used for training. By reducing spatial autocorrelation between the training and test sets, this strategy provided a more realistic estimate of error under cross-area deployment conditions.

#### 2.6.4 Uncertainty reporting

To support risk-aware decision-making in forest management, both point predictions and prediction intervals (PIs) were constructed and their empirical coverage was evaluated under the cross-validation framework.

Within the same outer cross-validation framework, conformal prediction intervals were constructed based on the absolute calibration residuals defined in Equation 20:

(20) $$r_{p}=|y_{p}-{\hat{y}}_{p}|$$

The (1α)-quantile of these residuals, denoted as $q_{1-\alpha }$, was then estimated (e.g., α=0.1 for a nominal 90% coverage rate), and used to construct a global conformal prediction interval according to Equation 21:

(21) $$PI_{p}=[\hat{y}-q_{1-\alpha },\;\hat{y}+q_{1-\alpha }]$$

Empirical coverage and mean interval width were subsequently evaluated on the corresponding test folds.

Because residual distributions often differ across plots of different quality levels, with lower-quality plots more prone to error inflation and under-coverage under a global interval, we further implemented Mondrian conformal prediction for quality-conditioned calibration. Specifically, QC_grade(High/Medium/Low) was used as the conditioning variable, and a separate residual quantile $q_{1-\alpha }^{\left(g\right)}$ was estimated within each quality grade g. This yielded quality-adaptive prediction intervals according to Equation 22:

(22) $$PI_{p}(g)=[{\hat{y}}_{p}-q_{1-\alpha }(g),\;{\hat{y}}_{p}+q_{1-\alpha }(g)]$$

This approach allows interval width to vary with data quality and can improve the empirical calibration of prediction intervals across quality grades. In this way, uncertainty reporting can support differentiated review triggering and risk control in deployment-oriented applications.

Because the plots were spatially structured, the exchangeability assumption underlying conventional conformal prediction cannot be regarded as automatically satisfied. In particular, spatial dependence between nearby plots may induce dependence between calibration and test residuals and can therefore affect the nominal coverage of standard conformal intervals. We therefore interpret the conformal prediction results as empirical coverage assessments under the specified cross-validation design rather than as evidence of unconditional finite-sample coverage under arbitrary spatial dependence. To further examine the sensitivity of predictive performance to spatial structure, spatial block cross-validation was conducted separately from the conformal calibration procedure (Section 2.6.3). The spatial-block results were used to assess model transferability under spatial extrapolation, whereas the conformal analysis was used to evaluate whether prediction intervals remained reasonably calibrated across plot-quality groups. This distinction avoids treating spatial block validation as a direct solution to the exchangeability requirement of conformal inference.

#### 2.6.5 Minimum plot requirement, gating, and quality-governance evaluation

To derive operationally relevant recommendations, three additional deployment-oriented evaluations were conducted within the main-model framework.

First, learning curves were used to quantify the minimum number of plots required for stable model performance. Training subsets were repeatedly sampled at increasing sizes, and for each subset the model was trained on the sampled plots and evaluated on the remaining plots. The mean and variability of RMSE were then summarized across repeated runs to provide an empirical range for the plot sample size required to achieve a target level of predictive accuracy.

Second, alternative quality-governance strategies were compared to characterize the trade-off among sample coverage, robustness, and extreme failure risk. Specifically, we compared: (i) a hard-exclusion strategy applied after plot-level consistency screening (QC123-hard), (ii) a full-retention strategy without plot-level exclusion (QC12), and (iii) a weighted-training strategy based on QC_score. Their effects on overall prediction error, stability, and extreme-error events were evaluated under the same cross-validation framework.

Third, to support deployment-oriented data admission, different QC_score gating thresholds were applied to the QC12 plot set to create scenarios with different retention rates, and cross-validated RMSE was evaluated under each threshold. A composite cost function was then constructed, for example:


Total cost=RMSE+drop rate

to identify operating points at which fewer plots were retained but prediction reliability was improved. This provided a direct quantitative basis for threshold selection under resource constraints and for the design of deployment-time gating strategies.

## 3 Results

### 3.1 Data screening and consistency checks

This study used 26,767 individual-tree records from 133 plots to examine data screening and consistency checks under real operational noise in UAV-LiDAR and field-inventory integration. Overall, quality control proceeded from the tree level to the plot level in a progressively stricter manner. The workflow first identified obvious risks of mismatching in tree-level spatial linkage, then addressed contamination from missing-value coding and physically implausible records, and finally examined the consistency between field-measured tree height and LiDAR-derived TreeHeight at the plot level to detect structural anomalies likely to affect subsequent modeling and application. The results showed that the main threats to data usability were not confined to scattered tree-level errors, but extended to structural inconsistency at the plot level.

At the basic screening stage, QC.1 mainly addressed inconsistencies in coordinate-field conventions, which are common in operational datasets. When matching distances were calculated in parallel under the original coordinate interpretation and the swapped X/Y interpretation, 35.97% of records showed a smaller distance under the swapped interpretation. This indicates that, without coordinate-consistency correction, tree matching would be subject to a substantial risk of systematic mismatch. After correction, the overall distribution of matching distance was relatively concentrated, with a median of 0.481 m and a 95th percentile of 2.835 m, although a few extreme outliers remained, with a maximum of approximately 32.17 km. Threshold sensitivity analysis further showed that when the matching-distance threshold was set to 3, 5, and 10 m, record-retention rates were 95.58%, 98.98%, and 99.78%, respectively, while plot coverage remained unchanged at 133 plots under all three thresholds. Balancing mismatch control against sample retention, the main analysis adopted a gating threshold of d≤5m, which retained 26,493 records, or 98.98% of the original dataset.

QC.2 was mainly used to address missing-code contamination and physically implausible records. In the raw data, there were 4 records with DBH ≤0, 12 records with H≤0, and 142 records in which crown base height violated physical constraints. In addition, the plot-level mean-height field contained 2,495 records in which “0” had been used to represent missingness, accounting for 9.32% of the original dataset. Although these problems represented only a small proportion of the overall data, they would directly contaminate response variables and structural predictors if allowed to enter plot aggregation and model training. We therefore adopted a conservative treatment in which only clearly erroneous values and confirmed coding anomalies were set to missing or flagged, without further intervention on valid observations. In the dataset after QC.1, anomaly flags introduced by QC.2 remained below 1% for all variables, indicating that this stage involved low-intensity cleaning but removed a small number of high-impact errors (**Figure 2**).

**Figure 2:**
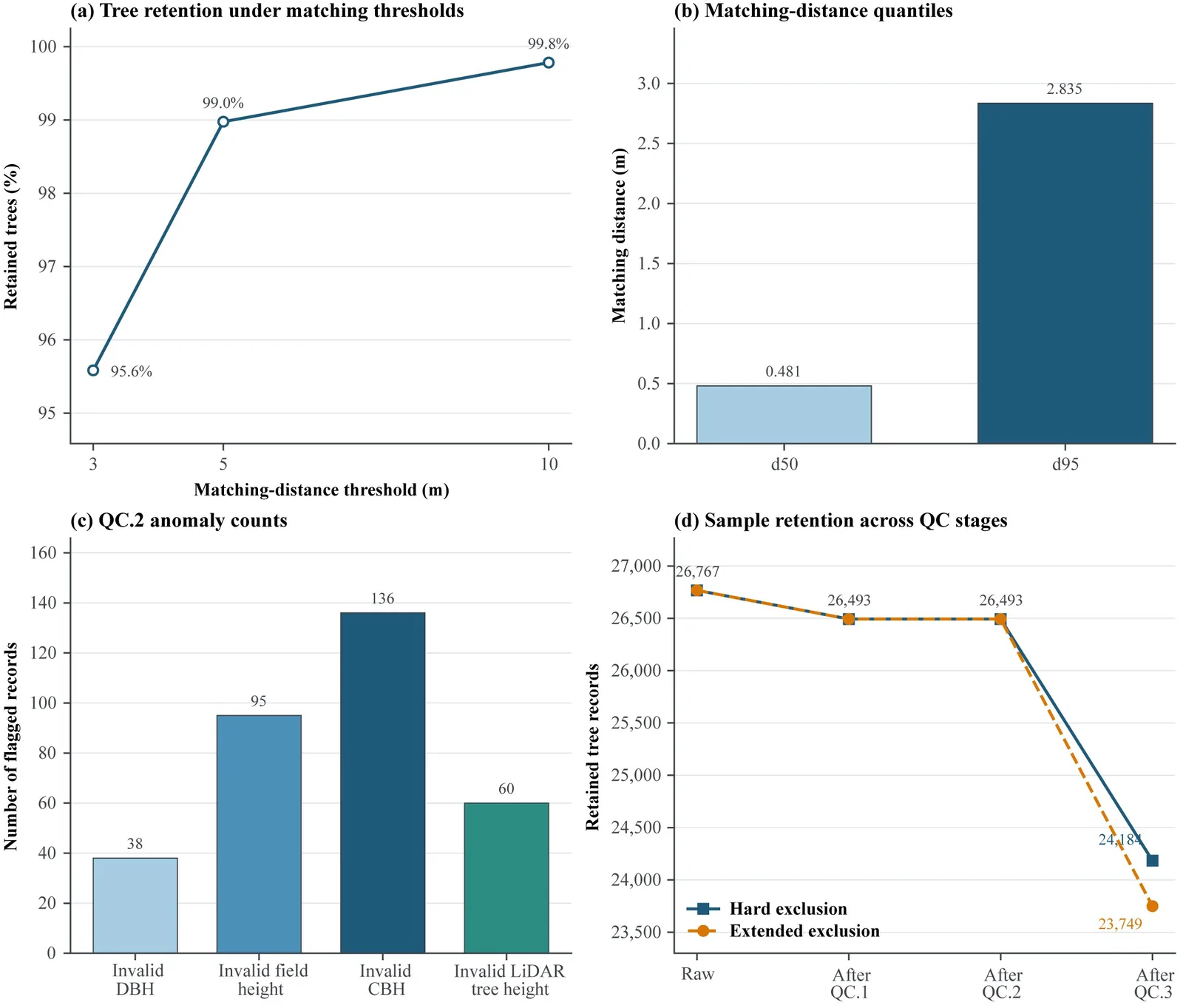
Basic data screening and sample retention across quality-control stages.

After tree-level screening, QC.3 shifted the focus to the plot scale and evaluated the consistency between field-measured tree height and LiDAR-derived TreeHeight. Overall, the 133 plots showed a relatively stable pattern of positive bias, with a median $\text{Δ}{\bar{H}}_{p}$ of +2.51 m, a mean of +2.94 m, a median $RMSE_{p}$ of 3.64 m, and a median $corr_{p}$ of 0.475. Even so, a small number of structurally abnormal plots were still evident. Based on robust thresholds, the anomaly threshold was 7.747 m for RMSE and 6.156 m for $|\text{Δ}\bar{H}|$. In total, 4 plots exceeded the RMSE threshold and 7 plots had $corr_{p}\leq 0$. Plot 80 was the most typical case, with an $RMSE_{p}$ of approximately 18.79 m and a $\text{Δ}{\bar{H}}_{p}$ of approximately 18.07 m, indicating severe distortion in the height measurement system. On this basis, the main analysis set, QC123-hard, excluded 11 plots in total, accounting for 8.27% of all plots; the number of plots was reduced from 133 to 122, and the number of tree-level records from 26,493 to 24,184. Notably, after excluding these anomalous plots, the median $\text{Δ}{\bar{H}}_{p}$ remained +2.36 m, indicating that the overall positive bias was not driven solely by a few extreme plots. The main role of QC.3 was therefore not to remove overall bias, but to identify and isolate structural anomalies that could undermine plot-level relationships. Under the stricter sensitivity set, QC123-full, plots with anomalous $|\text{Δ}\bar{H}|$ were further excluded, resulting in the removal of 15 plots in total and retention of 118 plots and 23,749 tree-level records. The overall conclusions remained unchanged. The plots flagged by the consistency check and their key diagnostic statistics are listed in **Supplementary Table 1**.

Beyond rule-based screening, matching reliability, consistency, and completeness were further integrated into a continuous quality indicator, QC_score, for plot-level risk stratification. Using high-error events in cross-validation as an external reference, the risk score 1−QC_score yielded an AUC of 0.557, indicating limited discriminative ability. This suggests that QC_score is better interpreted as a prioritization tool for plot review and admission rather than a high-precision anomaly classifier. Even so, when plots were grouped by QC_score terciles, the proportion of high-error events increased from 15.56% in the high-quality group to 25.00% and 34.09% in the medium- and low-quality groups, respectively. This indicates that QC_score retained value as a coarse but useful ranking signal for plot-level review priority. This indicates that QC_score still provided a meaningful signal for ranking plot-level risk. Overall, these results show that the main quality problems in UAV-LiDAR and field-inventory integration arise not only from tree-level noise, but also from structural inconsistency at the plot scale. Data screening should therefore extend beyond record-level cleaning to include plot-level consistency checks and quality stratification (**Figure 3**).

**Figure 3:**
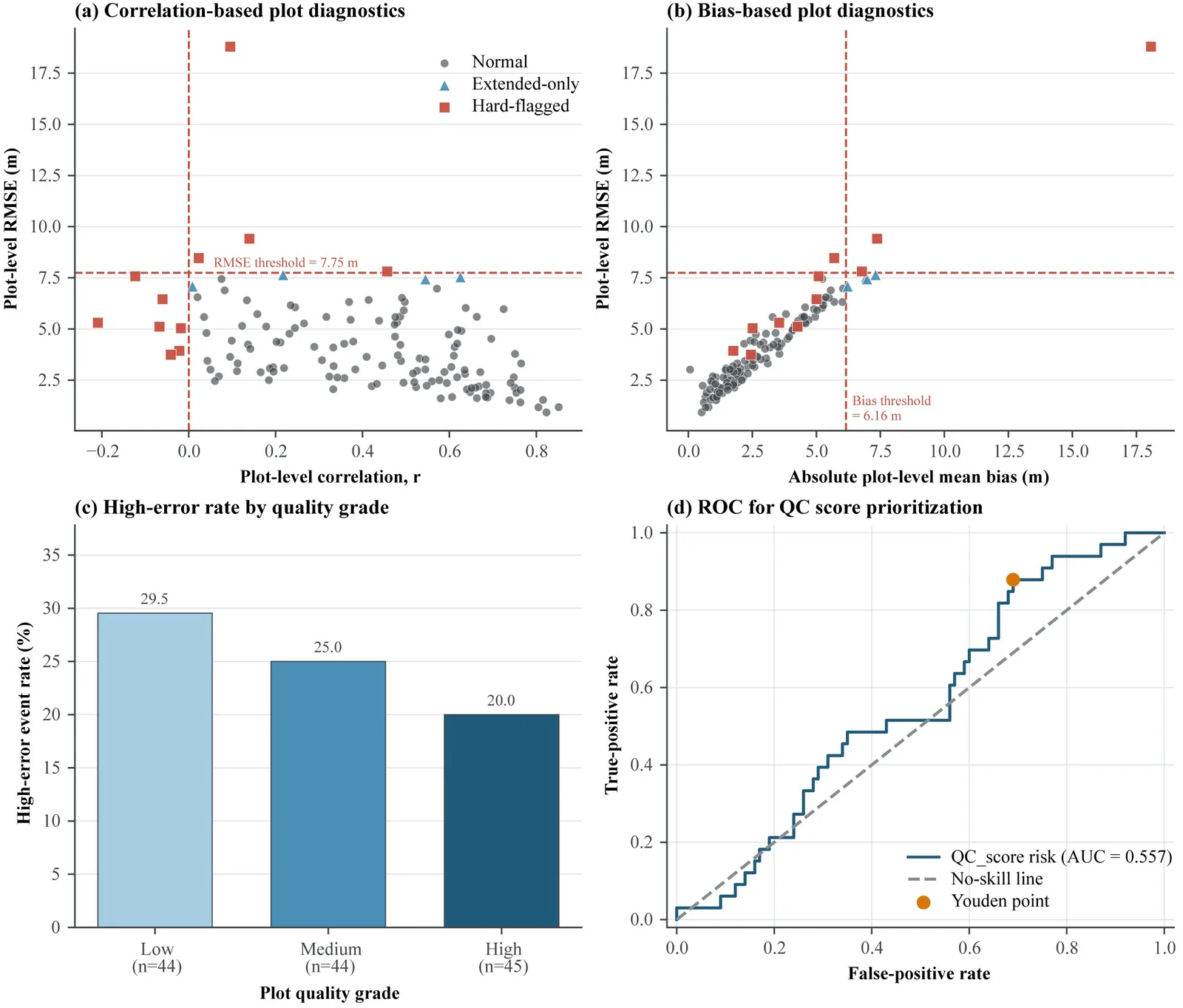
Plot-level consistency diagnostics and QC_score-based risk stratification.

### 3.2 Model performance under different quality-control strategies

To evaluate the effects of quality control on plot-level prediction, four data scenarios were compared under a consistent modeling framework: QC.1, which applied only matching-quality gating; QC.12, which further addressed coding anomalies and physically implausible records; and QC123-hard and QC123-full, which additionally incorporated plot-level consistency screening. The response variables were mean plot height and dominant plot height, and model performance was evaluated using repeated cross-validation based on RMSE, MAE, $R^{2}$, and bias. The results showed that applying QC.2 alone did not lead to a stable improvement in model performance, whereas a clearer gain in both accuracy and stability emerged when quality control was extended to the plot scale. In other words, the main factor limiting model performance was not simply the presence of local erroneous records, but the plot-level structural inconsistency that remained after tree-level cleaning (**Figure 4**). This also explains why QC.3 should not be regarded as an optional additional screening step, but rather as a key component in achieving stable operational performance.

**Figure 4:**
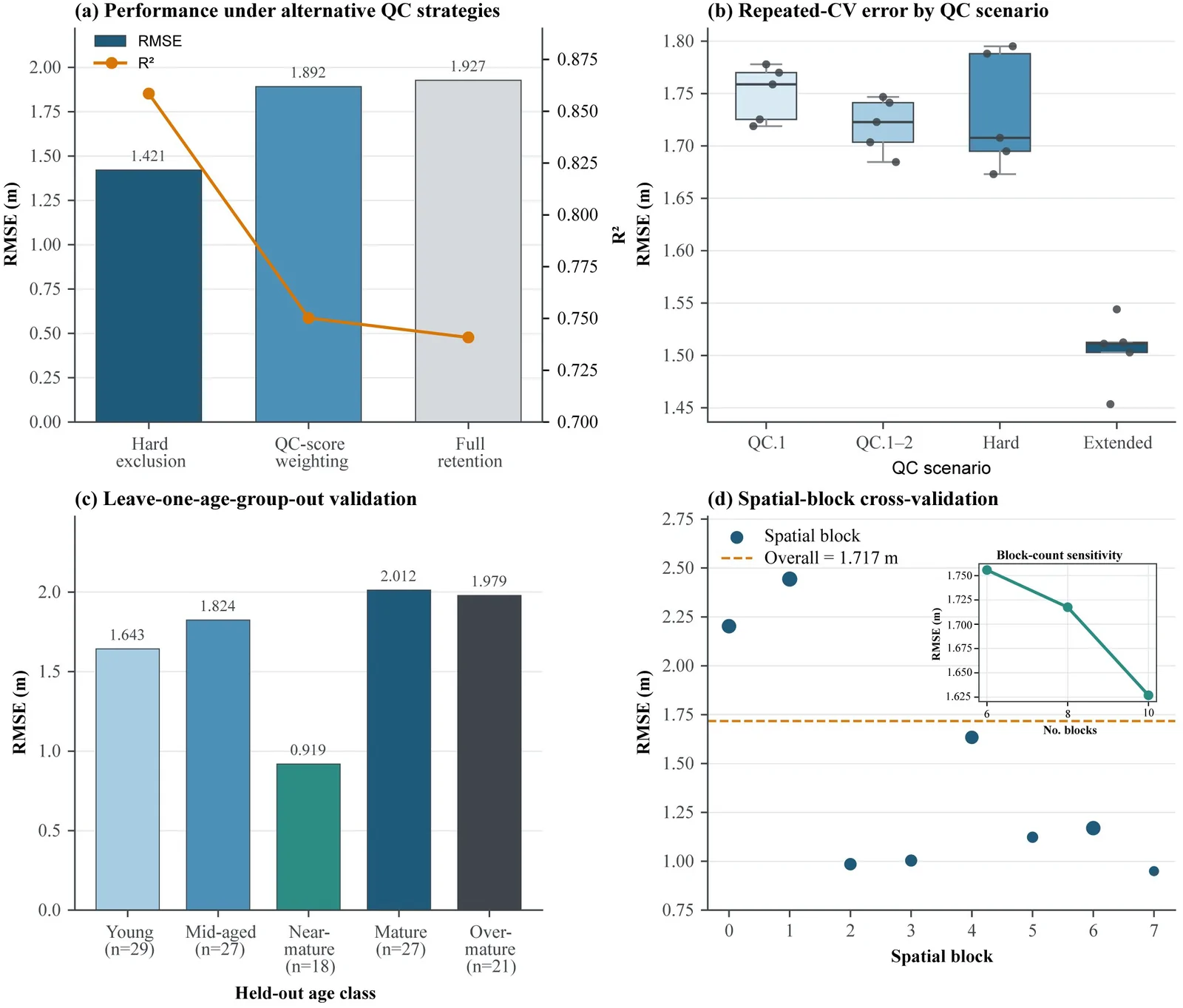
Model performance under alternative quality-control strategies and extrapolation settings.

Using QC123-hard as the main analysis set, we further compared different model families and feature-set combinations. Overall, the comparison among ridge regression, GAM, and GBDT showed that ridge regression provided the best balance among predictive accuracy, stability, and interpretability, and was therefore selected as the main model for subsequent analyses. Detailed results for all model-family and feature-set combinations are reported in **Table 2**. For dominant plot height, the best-performing configuration was ridge regression with F2, which combined LiDAR-derived structural features with topographic variables and achieved an RMSE of 1.447±0.024m and an $R^{2}$ of 0.853. For mean plot height, the best-performing configuration was ridge regression with F3, which further incorporated management and background variables, yielding an RMSE of 1.307±0.034m and an $R^{2}$ of 0.856. These results also indicate that LiDAR-derived structural features explained most of the model variance, that topographic variables provided additional but secondary value, and that management-related variables contributed only limited gains, with a modest but relatively consistent improvement observed for mean plot height. Permutation importance further showed that metrics related to canopy-height distribution were the dominant predictors, whereas the contribution of topographic variables was comparatively smaller. Taken together, ridge regression was not the most complex model, but it produced more stable and more interpretable results, and was therefore better aligned with the requirements of auditability and recalibratability in operational forest inventory.

**Table 2:** Comparison of model families and feature sets for the two plot-level height targets.

Target	Model	Feature set	RMSE mean (m)	RMSE SD (m)	R2 mean	R2 SD
H_dom20_field	GAM	**F1**	1.955	0.204	0.730	0.059
H_dom20_field	GAM	**F2**	1.759	0.204	0.781	0.054
H_dom20_field	GAM	**F3**	220.772	30.740	-3464.818	994.694
H_dom20_field	GBDT	**F1**	1.833	0.073	0.765	0.019
H_dom20_field	GBDT	**F2**	1.732	0.056	0.790	0.014
H_dom20_field	GBDT	**F3**	1.630	0.039	0.814	0.009
H_dom20_field	Ridge	**F1**	1.466	0.027	0.849	0.006
H_dom20_field	**Ridge**	**F2**	**1.447**	**0.024**	**0.853**	**0.005**
H_dom20_field	Ridge	**F3**	1.468	0.029	0.849	0.006
H_mean_field	GAM	**F1**	1.698	0.134	0.756	0.040
H_mean_field	GAM	**F2**	1.611	0.089	0.781	0.025
H_mean_field	GAM	**F3**	212.673	28.361	-3864.713	1086.667
H_mean_field	GBDT	**F1**	1.608	0.075	0.782	0.020
H_mean_field	GBDT	**F2**	1.526	0.081	0.803	0.021
H_mean_field	GBDT	**F3**	1.481	0.076	0.815	0.019
H_mean_field	Ridge	**F1**	1.340	0.033	0.849	0.007
H_mean_field	Ridge	**F2**	1.339	0.031	0.849	0.007
H_mean_field	**Ridge**	**F3**	**1.307**	**0.034**	**0.856**	**0.008**

F1 includes LiDAR-derived predictors only; F2 adds topographic variables; F3 further adds management-related variables. Values are reported as mean ± SD across repeated cross-validation runs. The GAM model with F3 showed numerical instability and is retained for transparency rather than as a deployable candidate.

Bold values indicate the selected model–feature set combination for each response variable based on overall predictive performance.

The optimal feature configuration differed between the two height metrics, which may reflect their different structural definitions. Dominant plot height represents the mean height of the tallest 20% of valid trees and therefore primarily characterizes the upper-canopy structure of the plot. The LiDAR-derived structural variables in F1 already capture information on the vertical height distribution and upper-canopy structure. Accordingly, adding topographic variables in F2 resulted in only a modest improvement in Ridge performance for dominant plot height, with RMSE decreasing from 1.466 to 1.447 m. The further addition of management and stand-background variables in F3 did not provide additional improvement, with RMSE increasing slightly to 1.468 m.

Mean plot height, in contrast, represents the average height across all valid trees in a plot and therefore describes broader stand-level structure rather than only the upper canopy. For Ridge regression, adding the topographic variables in F2 produced almost no change in predictive performance, with RMSE decreasing from 1.340 to 1.339 m. However, the addition of management and stand-background variables in F3 further reduced RMSE to 1.307 m and increased R² from 0.849 to 0.856. These results indicate that the marginal contribution of additional predictors was response-specific: LiDAR-derived structural variables provided the primary predictive information for both height metrics, whereas contextual variables provided additional information mainly for mean plot height.

To further examine whether deployment error could be reduced through simple linear calibration, we fitted a robust linear regression between LiDAR-derived TreeHeight and field-measured tree height within each training fold and rebuilt the models after calibration. This step did not yield meaningful improvement. On the contrary, for dominant plot height prediction, the calibrated model showed a slight increase in RMSE from 1.732±0.056m to 1.761±0.080m, while $R^{2}$ decreased from 0.790 to 0.782 and bias did not show clear convergence (**Table 3**). This suggests that the main source of model error was not a global scale offset that could be corrected through a single linear relationship, but rather the combined effects of plot-level inconsistency in measurement systems and structural noise. Compared with *post hoc* calibration, early identification and treatment of problematic plots was therefore more effective and more consistent with the logic of deployment-oriented quality control (**Figure 5**).

**Table 3:** Operational evaluations under three distinct scenarios.

3a. QC_score-based plot admission on the QC12 dataset							
QC_score threshold	Retained plots (n)	Retention rate		Drop rate	RMSE	R2	Total cost
**0.066**	133	1.000		0.000	1.927	0.741	1.927
**0.361**	126	0.947		0.053	1.877	0.747	1.930
**0.413**	120	0.902		0.098	1.971	0.715	2.069
**0.473**	114	0.857		0.143	1.865	0.751	2.008
**0.496**	107	0.805		0.195	1.432	0.859	1.628
**0.531**	101	0.759		0.241	1.420	0.851	1.661
**0.579**	**95**	**0.714**		**0.286**	**1.146**	**0.907**	**1.432**
**0.614**	89	0.669		0.331	1.163	0.908	1.494
**0.635**	82	0.617		0.383	1.106	0.909	1.490
**0.655**	76	0.571		0.429	1.118	0.903	1.546
**0.672**	70	0.526		0.474	1.159	0.89	1.633
**0.703**	64	0.481		0.519	1.143	0.899	1.662
3b. Quick-repair sensitivity under different calibration sample sizes							
Scenario		Calibration sample size (m)			RMSE_mean (m)		RMSE SD (m)
**QC12 (baseline)**		—			2.597		1.42
**QC123-hard**		—			1.459		0.476
**Quick repair (flagged)**		5.0			1.481		0.115
**Quick repair (flagged)**		10.0			1.389		0.057
**Quick repair (flagged)**		20.0			1.94		1.218
3c. Quality-adaptive uncertainty across plot quality grades (nominal coverage = 90%)							
Method	QC grade	n	Coverage		Avg. PI width (m)	Median PI width (m)	Calibration quantile q
**Global**	Low	41	0.829		5.365	5.365	—
**Mondrian**	Low	41	0.902		4.143	4.143	2.071
**Global**	Medium	40	0.9		5.365	5.365	—
**Mondrian**	Medium	40	0.9		4.123	4.123	2.061
**Global**	High	41	0.951		5.365	5.365	—
**Mondrian**	High	41	0.902		3.281	3.281	1.64

In **Table 3a**, total cost was defined as cost_err × RMSE + cost_drop × drop rate, with cost_err = 1 and cost_drop = 1. In **Table 3b**, quick repair was evaluated on plots flagged for review. In **Table 3c**, prediction intervals target 90% nominal coverage; the calibration quantile q applies to the Mondrian method only.

Bold values indicate the selected QC_score operating point that minimized the composite cost function defined as cost_err × RMSE + cost_drop × drop rate, with cost_err = 1 and cost_drop = 1.

**Figure 5:**
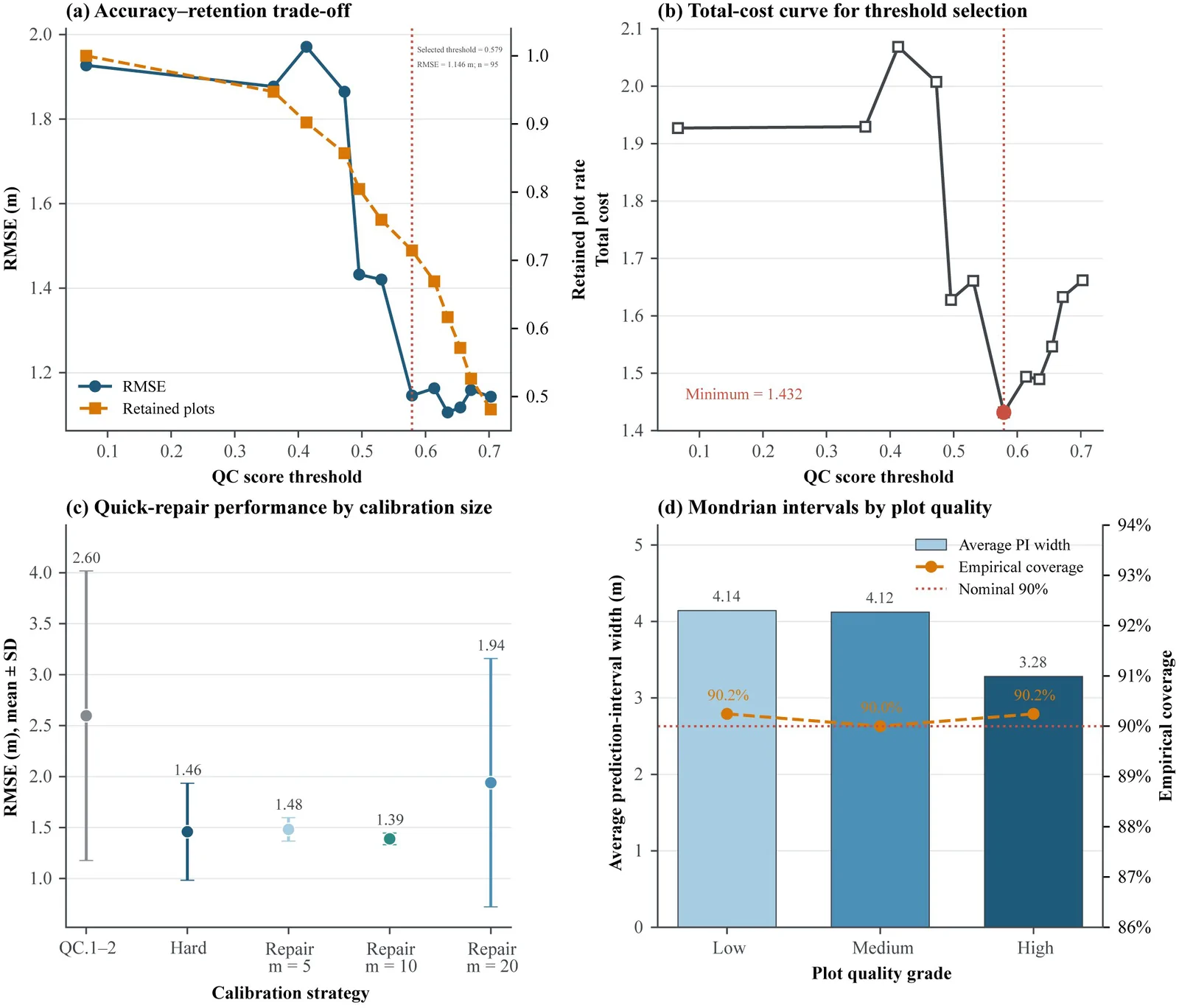
Operational use: threshold gating, quick repair, and quality-adaptive uncertainty.

Overall, these results support two main conclusions. First, although basic data cleaning is necessary, its main value lies in removing obvious errors; what ultimately determines model performance is whether plot-level consistency has been adequately controlled. Second, once this condition is met, a model with a clear structure, low maintenance cost, and strong interpretability is more appropriate for the present study than one that simply pursues greater functional complexity. Accordingly, all subsequent analyses were conducted using QC123-hard as the main analysis set and ridge regression as the main model, so that gating, repair, and uncertainty evaluation were all based on a relatively stable and interpretable foundation.

## 4 Discussion

### 4.1 Interpreting plot-level error and model behaviour

The clearest pattern emerging from this study was that isolated tree-level errors and plot-level structural inconsistency did not have equivalent consequences for model performance. QC.1 and QC.2 identified problems such as coordinate misinterpretation, incorrect missing-value codes, and physically implausible measurements. These errors required correction because they could affect tree matching and the calculation of plot metrics. Nevertheless, errors confined to a small number of tree records may be partly attenuated when observations are aggregated to the plot level. By contrast, a systematic mismatch affecting an entire plot changes the relationship between field measurements and LiDAR-derived structure and therefore has a more direct influence on model fitting and prediction.

Previous forest-inventory studies have mainly examined errors associated with individual-tree delineation, field-reference measurements, positional uncertainty, and the co-registration of field and remotely sensed data. The present results are consistent with the broader finding that reference-data errors can propagate into remote-sensing models, but they also show that the effect depends on the scale at which the error occurs. A plot may contain no obviously invalid tree records and still be inconsistent with the corresponding LiDAR observations. This distinction explains why conventional record-level screening alone was insufficient: it verified whether individual measurements were plausible, but not whether the two measurement systems described the same plot structure.

QC.3 addressed this remaining source of error by evaluating structural consistency at the same spatial scale as the inventory predictions. Its stronger effect on prediction accuracy suggests that plot-level discrepancies cannot always be resolved by increasing model complexity or applying a common statistical correction. When a discrepancy is systematic within a plot, a fitted model may treat it as part of the response–predictor relationship rather than as measurement error. Such plots can therefore influence both coefficient estimation and model evaluation, particularly when the available sample is limited. In operational applications, plot-level quality control should consequently be treated as part of model validation and data admission, rather than solely as a preliminary data-cleaning step.

The contribution of additional predictor groups also depended on the response variable. Dominant plot height was calculated from the tallest 20% of valid trees and therefore mainly represented upper-canopy structure. The LiDAR variables in F1 already described much of this information through canopy-height distributions and upper-canopy characteristics. Adding topographic variables in F2 provided a modest improvement, whereas the management and background variables added in F3 contributed little further information. Mean plot height, in contrast, represented the full set of valid trees and was more closely related to overall stand development. Variables describing management and stand background could therefore provide information that was not fully represented by LiDAR structure or topography alone, which helps explain the better performance of F3 for this response. The differences among the feature sets were not large, and should not be interpreted as evidence that one predictor group is universally preferable. Rather, they indicate that the value of contextual variables depends on the component of stand structure being predicted.

### 4.2 Why plot-level inconsistency matters more than tree-level noise

The results further indicate that plot-level structural inconsistency deserves more attention than local error associated with individual trees. When only QC.1 and QC.2 were applied, model improvement was limited; once QC.3 was introduced, gains in both accuracy and stability became much more evident. This suggests that, in practice, model performance is often degraded not by a small number of mismatched trees, but by plots that contain systematic bias at the aggregate level. Local noise typically increases error variance, whereas structural inconsistency can directly alter plot-level relationships and cause the model to learn misleading patterns.

Plot 80 provides a clear example. This plot did not show unusual matching distance at the tree level, yet severe distortion existed between field-measured tree height and LiDAR-derived TreeHeight, ultimately leading to a very large prediction error at the plot level. This demonstrates that normal matching distance alone is not sufficient to judge whether a plot is suitable for training or deployment. For forest monitoring, greater attention should be paid to plots that appear acceptable on the surface but are structurally distorted, because they are more easily overlooked by routine cleaning while posing greater risk once the model is applied.

This also helps explain why hard exclusion was more effective than simple soft weighting in the comparisons conducted here. When anomalies consist mainly of mild noise or local errors, reducingtheir contribution through weighting may be reasonable because the underlying relationships remain largely intact. For structurally inconsistent plots, however, the source of error dmay differ systematically from that of the majority of plots, and simple down-weighting may not be sufficient to prevent their influence on model fitting.

The present study did not systematically compare QC-based exclusion with a broader range of robust regression approaches, such as Huber-type loss functions or other outlier-resistant estimators. Such methods provide an important alternative because they can reduce the influence of anomalous observations while retaining them in the analysis. Accordingly, the evidence presented here should not be interpreted as demonstrating that hard exclusion is universally superior to statistical adjustment. Rather, within the quality-governance strategies evaluated in this study, QC123-hard provided greater improvement in predictive stability than the simple weighted-training strategy applied to the same data. For operational forest inventory, this finding suggests that plots showing clear structural inconsistency should at least be subjected to formal review, while robust regression and other outlier-resistant approaches remain promising alternatives for future evaluation. This distinction is important because exclusion and robust statistical adjustment address the same problem through different mechanisms: the former controls data admission, whereas the latter attempts to reduce the statistical influence of observations that are retained.

At the same time, this does not mean that all problematic plots must be removed. The quick-repair results indicate that, for some bias-dominated anomalies, local correction based on a small number of sample trees can still be useful, particularly when m=10, where performance remained relatively stable. Overall, however, this approach is better regarded as a low-cost corrective option than as a universal repair strategy. For plots in which structural relationships are already substantially distorted, review or reprocessing remains the more reliable choice.

### 4.3 Implications for forest inventory and uncertainty reporting

From the perspective of forest inventory and resource monitoring, the contribution of this study lies not simply in comparing different modeling strategies, but in proposing a workflow that is closer to operational needs. For a new batch of data, obvious problems should first be identified through basic quality checks so that low-level errors do not enter plot aggregation and model training. Structural consistency should then be examined at the plot level, so that plots most likely to undermine model performance can be identified in advance. The purpose of this process is not merely to make the dataset appear cleaner, but to improve the usability and credibility of the final outputs.

QC_score nevertheless has practical value as a coarse ranking measure rather than as a stand-alone anomaly classifier. Although its AUC of 0.557 indicates limited discrimination at the individual-plot level, the higher incidence of prediction errors among lower-quality plots suggests that the score retains some information for prioritizing review and managing plot admission when inspection resources are limited. It should therefore be used for preliminary triage in combination with the underlying quality diagnostics, rather than as the sole basis for automatic plot exclusion. Similarly, the selected threshold of 0.579 represents a cost-sensitive operating point derived from the present dataset. It reflects the specific trade-off between prediction error and plot retention and should be recalibrated when forest conditions, acquisition protocols, or operational costs differ.Another important implication concerns uncertainty reporting. Prediction uncertainty should not be handled uniformly across all plots. If all plots are assigned the same type of global interval, high-quality plots are likely to receive unnecessarily wide intervals, whereas low-quality plots may suffer from inadequate coverage. The present results show that after calibration within quality strata, coverage becomes more balanced across plot groups and higher-quality plots receive narrower intervals. This suggests that uncertainty reporting should not be treated simply as an additional statistical output, but as part of the interpretation of plot-level results. For high-quality plots, point estimates together with relatively narrow intervals may support direct use; for lower-quality plots, wider intervals imply that more cautious interpretation is needed; and for structurally anomalous plots, review remains a more appropriate response than relying on wide intervals as a substitute for quality control.

The additional evaluation of learning curves, cross-age validation, and spatial block cross-validation further helps define the boundary of model applicability. Random validation often yields relatively optimistic estimates, whereas error increased clearly under spatial extrapolation, indicating that real deployment conditions are usually more demanding than standard cross-validation settings. Evaluation of UAV-LiDAR-assisted forest inventory should therefore not rely solely on average accuracy under random validation, but should also consider extrapolation tests that better reflect operational scenarios, together with risk control based on plot-level quality stratification. Only models that remain reasonably robust under such conditions are likely to be useful in practice.

### 4.4 Limitations and future work

The framework proposed here should be understood as an application-oriented quality-control approach rather than as a fixed parameter template. First, the main value of QC_score lies in risk ranking and priority management rather than in high-precision classification. Future work should therefore consider incorporating additional operational metadata, batch information, or device-related differences to improve its discriminative ability. Second, gating thresholds will inevitably depend on study-area characteristics, forest structure, cost preferences, and application objectives. The threshold reported here is therefore most appropriately interpreted in relation to the present dataset and should not be treated as a universal standard.

In addition, although quick repair showed some promise for bias-dominated anomalies, its scope remains limited. In particular, when structural relationships are already strongly distorted, local correction based on a small number of sample trees cannot fully replace review or remeasurement. Future work could explore more stable low-cost repair strategies by combining field review information, batch-level correction information, and richer plot background variables. Finally, this study was conducted in Chinese fir plantations in Guangdong Province. Although the framework performed well in the present setting, its applicability to other forest types, inventory systems, and more complex terrain conditions remains to be tested. Further studies across a wider range of regions and data sources will be needed to assess the robustness of this approach and its potential for long-term monitoring and routine operational use.

## 5 Conclusion

This study shows that, in forest monitoring based on the integration of UAV-LiDAR and field inventory, the key factor affecting the reliability of plot-level results is not mainly scattered tree-level error, but structural inconsistency at the plot level. Basic data screening can identify problems such as inconsistent coordinate conventions, contamination from missing-value coding, and clearly implausible records. More critical, however, are those plots that appear acceptable at the individual-tree level but already exhibit inconsistencies in measurement systems at the plot scale. Although such plots are relatively few, they can exert disproportionate influence on model training and result interpretation. Plot-level consistency checks should therefore be regarded as a central component of quality control in forest monitoring.

Further analysis indicates that, once a plot is identified as structurally anomalous, within the quality-governance strategies evaluated in this study, hard exclusion was more effective than simple statistical weighting for the structurally inconsistent plots identified by QC.3. For some anomalies dominated by systematic bias, local correction based on a small number of sample trees shows potential as a low-cost option, but this approach should be regarded as supplementary rather than as a substitute for review or reprocessing of structurally inconsistent plots. At the same time, prediction outputs should not be interpreted uniformly across all plots. Instead, uncertainty should be expressed in a way that reflects plot quality, so that higher-quality plots can be used with clearer decision boundaries, whereas lower-quality plots are interpreted more cautiously.

Overall, the contribution of this study lies not only in proposing a data-processing workflow, but in clarifying how integrated forest-monitoring outputs should be used in practice. Some plots can be admitted directly for analysis and delivery, some should be prioritized for review, and some results should be interpreted only under wider uncertainty bounds. By linking plot screening, anomaly treatment, and uncertainty-aware reporting, UAV-LiDAR-assisted forest inventory can move beyond simple prediction outputs toward monitoring information that is more traceable, auditable, and useful for management.

## Data Availability

The raw data supporting the conclusions of this article will be made available by the authors, without undue reservation.
