# Supplementary material for: A deployment-oriented quality-control framework for integrating UAV LiDAR and field inventory in precision forestry
Source: Front Plant Sci. 2026 Sep 14;17:1932005. doi: 10.3389/fpls.2026.1932005 (PMC13616950; doi:10.3389/fpls.2026.1932005)
Supplement: Supplementary file 1 [file Table1.docx]

Supplementary Table 1. Plots flagged by the QC.3 consistency check and their key diagnostic statistics.

| **Plot ID** | **Mean height difference (m)** | **Plot RMSE (m)** | **Plot correlation (r)** | **Valid matched pairs (n)** | **Triggering criterion** | **QC.3 exclusion set** |
| --- | --- | --- | --- | --- | --- | --- |
| 5 | 4.269 | 5.114 | -0.067 | 229 | non-positive correlation | QC123-hard and QC123-full |
| 8 | 7.314 | 7.643 | 0.217 | 71 | absolute-bias threshold | QC123-full only |
| 9 | 6.948 | 7.532 | 0.625 | 140 | absolute-bias threshold | QC123-full only |
| 16 | 6.982 | 7.433 | 0.545 | 124 | absolute-bias threshold | QC123-full only |
| 52 | 1.757 | 3.932 | -0.022 | 245 | non-positive correlation | QC123-hard and QC123-full |
| 63 | 3.549 | 5.305 | -0.209 | 367 | non-positive correlation | QC123-hard and QC123-full |
| 64 | 5.088 | 7.576 | -0.123 | 191 | non-positive correlation | QC123-hard and QC123-full |
| 68 | 6.223 | 7.082 | 0.008 | 94 | absolute-bias threshold | QC123-full only |
| 80 | 18.069 | 18.795 | 0.095 | 366 | RMSE threshold; absolute-bias threshold | QC123-hard and QC123-full |
| 84 | 5.701 | 8.465 | 0.023 | 251 | RMSE threshold | QC123-hard and QC123-full |
| 95 | 6.779 | 7.811 | 0.457 | 144 | RMSE threshold; absolute-bias threshold | QC123-hard and QC123-full |
| 96 | 2.511 | 5.036 | -0.018 | 129 | non-positive correlation | QC123-hard and QC123-full |
| 108 | 7.374 | 9.411 | 0.140 | 136 | RMSE threshold; absolute-bias threshold | QC123-hard and QC123-full |
| 120 | 5.008 | 6.456 | -0.060 | 158 | non-positive correlation | QC123-hard and QC123-full |
| 122 | 2.445 | 3.743 | -0.041 | 75 | non-positive correlation | QC123-hard and QC123-full |

Notes: Mean height difference = mean(LiDAR-derived tree height − field-measured tree height); positive values indicate higher LiDAR-derived heights. QC123-hard flags plots when plot RMSE > 7.747 m, correlation ≤ 0, or the number of valid matched pairs < 5. QC123-full applies these criteria and additionally flags plots when the absolute mean height difference > 6.156 m. Thresholds were calculated as the median + 3 × MAD. No plot was flagged for fewer than five valid matched pairs in this dataset.
